# Passing Events and Topics

**Published:** 1921-03-19

**Authors:** 


### Passing Events and Topics.

### Finsen-Light Centres for London.

The London Hospital has asked the Bethnal Green Borough Council to undertake the responsibility for the payment of certain fees for the treatment of local residents attending the hospital for the treatment of lupus by means of the Finsen light. The authorities of the City of London Hospital for Diseases of the Chest are willing to arrange for the treatment of Bethnal Green lupus patients at the London Hospital, such treatment being ancillary to the tuberculosis dispensary scheme. In the circumstances the Health Committee recommends arrangements with the City of London Hospital for the provision of Finsen-light treatment for cases of lupus at the London Hospital at an estimated cost of £10 for the first half-year.

### A Joint Rural Scheme.

Essex County Council has prepared a scheme for dealing with the maternity and child-welfare work in twenty-five rural districts having a total population of 208,000. It is proposed in the first place to start child-welfare centres where school clinics are now held, so that the same premises, staff, and, to some extent, the equipment, can be used for the two purposes. Such centres could be established and maintained at a minimum expense of about £20 each per annum. Existing welfare centres would be assisted and supervised by the County Council, and grants would be made to the local committees not exceeding £20 per annum per centre. Attached to each centre would be a local committee composed of members of the County Council, District Education Sub-Committee, local sanitary authorities, and voluntary associations or persons interested in child welfare; and a grant of £20 might be made to each committee. The total cost of the scheme for the twenty-five districts would be £3,000, of which one-half would be paid by the State.

### London School of Tropical Medicine.

Lord Milner's fund for the London School of Tropical Medicine now amounts to the substantial sum of £117,000. It will be remembered that the Red Cross Society gave £100,000 for the purchase and equipment of a school and hospital in Endsleigh Gardens, and the object of the present appeal is to raise £150,000 for maintenance and the prosecution of research.

### Books for Hospitals.

Mr. Edmund Gosse presided at a meeting in support of the British Red Cross Hospital Library. This organisation, started during the war for our soldiers and sailors, now deals with the reading needs of the civilian sick in hospitals and convalescent homes. Well-chosen books will be gratefully received at 48 Queen's Gardens, Lancaster Gate, W. 2.

### Reduction in Hospital Cost

The authorities of the National Hospital for the Paralysed and Epileptic, Queen Square, have intimated to the Ministry of Pensions that they are prepared to receive a reduced capitation grant for the ex-Service men at Lonsdale House, Clapham Park, the cost per patient having fallen to 7s. 6d. per day.

### Comedy at St. Mary's Hospital.

On the evening of March 11 a performance was given by the Musical and Dramatic Society of St. Mary's Hospital in the School Library, when "The Marriage of Kitty," by Cosmo Gordon-Lennox, was given, preceded by "Postal Orders," by Roland Pertwee. Both plays were exceedingly well presented, and were much appreciated by the audience which packed the hall. A special performance was also given to which all the nursing staff of the hospital were invited.

